# The effects of lateral pharyngoplasty on carotid intima-media thickness in patients with obstructive sleep apnoea

**DOI:** 10.5935/1984-0063.20220028

**Published:** 2022

**Authors:** Érika Pérez Iglesias, Michel Burihan Cahali

**Affiliations:** 1 Hospital do Servidor Público Estadual de São Paulo, Department of Otolaryngology - São Paulo - Brazil.; 2 Universidade de São Paulo, Department of Otolaryngology - São Paulo - Brazil.

**Keywords:** Sleep Apnea, Obstructive, Atherosclerosis, Carotid Intima-Media Thickness, Blood Pressure

## Abstract

**Introduction:**

Obstructive sleep apnea (OSA) is a known risk factor for development of carotid atherosclerosis. The treatment of OSA, through positive pressure devices or surgical procedures, may reduce the signs of subclinical atherosclerosis in apneic patients.

**Objective:**

The decrease of carotid intima-media thickness (CIMT) after treatment of OSA remains a highly controversial issue. Our purpose is to compare CIMT, which represents an early sign of atherosclerosis, before and at least 6 months after lateral pharyngoplasty in patients with OSA.

**Material and Methods:**

A total of 17 patients with OSA who underwent lateral pharyngoplasty were submitted to common carotid Doppler ultrasonography, 24-hour ambulatory blood pressure monitoring and type-1 polysomnography before and at least 6 months after surgery.

**Results:**

The median apnoea-hypopnoea index decreased from 22.6 to 5.9 (p<0.001). There were significant improvements in the arousal index, minimum oxyhaemoglobin saturation, Epworth sleepiness scale and reported snoring intensity. The surgical success rate (Shers criteria) obtained with the procedure was 76.4%. There was no significant variation in the mean CIMT after surgeries (right carotid artery, mean, 0.67 and 0.72 mm; left carotid artery, mean, 0.69 and 0.70 mm, pre- and postoperative, respectively, both p>0.05). Blood pressure measurements also did not significantly change.

**Conclusion:**

Notwithstanding a significant improvement in OSA after lateral pharyngoplasty, there was no significant reduction in CIMT in a follow-up of 6 months.

## INTRODUCTION

Evidence shows that obstructive sleep apnoea (OSA) is an independent risk factor for cardiovascular and cerebrovascular morbidity^1-3^. While the mechanisms that relate OSA to cardiovascular diseases are not yet fully understood, endothelial dysfunction, oxidative stress, chronic inflammation and sympathetic activation seem to play key roles in this process^4-6^.

Atherosclerosis, a chronic inflammatory disease characterized by the formation of atheroma plaques in blood vessels, is one of the direct causes of cardiovascular diseases, such as acute myocardial infarction and cerebrovascular accident^7,8^. Previous studies have demonstrated an independent relationship between OSA and atherosclerosis^8,9^. Most of these studies evaluated atherosclerosis by means of its subclinical signs, mainly the carotid intima-media thickness (CIMT), which shows increased endothelial thickness in OSA patients compared to healthy controls^8-13^. Whether an appropriate OSA treatment is able to reverse some of this endothelial thickness is highly controversial.

Continuous positive airway pressure (CPAP) is recognized as the first-line therapy for OSA. Previous studies have shown conflicting results on the effect of CPAP on CIMT in patients with OSA^14-16^. Compared to a control group, CPAP had no impact on CIMT in a meta-analysis of two randomized control trials, but the duration of CPAP treatment and the severity of OSA were independently associated with CIMT decrease in CPAP subgroups^16^. The authors of this meta-analysis could only hypothesize on the role of a higher baseline CIMT influencing the outcomes of CPAP therapy on CIMT^16^. On the other hand, another study showed that, even in patients with normal CIMT, OSA treatment with CPAP reduced CIMT after 2 years of therapy^17^. Two previous studies evaluated the impact of uvulopalatopharyngoplasty for OSA on the signs of atherosclerosis, including measures of CIMT, with conflicting results^18,19^. We aim to evaluate the impact of lateral pharyngoplasty on CIMT in patients with OSA.

## MATERIAL AND METHODS

This research project was approved by the ethics committee of our institution and was enrolled in the Brazil research platform under the number CAAE: 17898613.4.0000.5463.

From August 2012 to April 2013, we prospectively and consecutively included adult patients with OSA (apnoea-hypopnoea index, AHI>5/h, habitual loud snoring, with or without excessive daytime sleepiness) who refused or failed treatment with CPAP or oral appliances, regardless of the severity of OSA or pharyngeal anatomy. All patients who agreed with the study protocol signed the informed consent form. Our exclusion criteria were: age above 70 years, body mass index (BMI)>40kg/m^2^, diagnosis of cerebrovascular disease, neurological disease, heart disease or renal failure, and the use of some medications that could alter the sleep architecture (such as benzodiazepines and neuroleptics). In addition, those with a diastolic pressure greater than 120mmHg were also excluded during initial assessment in the office setting or during preoperative evaluations. Patients with controlled hypertension (in office measurement) were not excluded.

We consecutively included 25 patients of both sexes in the study protocol. Eight patients did not perform the postoperative evaluations and 17 completed our protocol. Patients with hypertension, diabetes and dyslipidaemia were instructed to keep using their same prescribed medications throughout the study.

Our protocol included evaluations with type-1 nocturnal polysomnography, 24-hour ambulatory blood pressure monitoring and carotid Doppler ultrasonography before and at least 6 months after the surgical procedure. All preoperative carotid Doppler and 24-hour ambulatory blood pressure monitoring (ABPM) were performed in the same week as the surgical procedure. The greatest time difference among postoperative ABPM, Doppler and polysomnography was 2 months. In addition, the following anthropometric measurements were performed: body mass index (BMI, the weight in kilograms divided by the square of height in metres, kg/m^2^) and neck circumference (measured horizontally at the mid thyroid cartilage) and waist circumference (measured horizontally at the top of the iliac crest), both with the patient standing.

The patients were classified into I-IV Friedman stage^20^. In addition, patients filled in the Epworth sleepiness scale^21^ for measuring excessive daytime sleepiness and self-reported their snoring intensity using a 1- to 10-point scale.

### Type-1 polysomnography

All patients underwent assisted nocturnal polysomnography in the same sleep laboratory, recording neurophysiological parameters (electroencephalogram, electro-oculogram, chin electromyogram, leg and mandibular electromyography), cardiorespiratory parameters (oral thermistor and nasal pressure cannula, electrocardiogram, pulse oximetry and measures of abdominal and thoracic respiratory effort), body position record, and snoring sensor. Apnoea was defined as a reduction of at least 90% in the airflow for at least 10 seconds. Hypopnoea was defined as a reduction of at least 50% in the nasal pressure cannula signal, for at least 10 seconds, associated with oxyhaemoglobin desaturation of 3% or more or to an arousal, according to the 2007 criteria of the American Academy of Sleep Medicine. Polysomnography was performed before and repeated at least 6 months after the surgeries.

The severity of OSA was classified based on the apnoea and hypopnoea index, considering as a normal value an AHI less than 5/h, mild OSA=AHI between 5 and 14.9/h, moderate OSA=AHI between 15 and 30/h and severe OSA=AHI greater than 30/h.

### Carotid ultrasonography

The CIMT was evaluated by an ultrasound study of the arterial system, carried out using a Philips model IE33 (Philips Medical Systems; Eindhoven, Netherlands), with a 10MHz high-resolution linear transducer. Two-dimensional ultrasound techniques, pulsed Doppler and Doppler with colour flow mapping were employed.

The examinations were performed in the radiology sector of our institution by the same experienced radiologist, who was blinded regarding the treatment status of the patient, with the patient awake in supine position. The intima-media thickness was measured by the software of the equipment on the right and left common carotid arteries, 1cm below the carotid bifurcation, at the region of greatest thickening. Values between 0.9 and 1.4mm represented abnormal thickening and those above 1.4mm were considered an atheroma plaque.

### 24-hour ambulatory blood pressure monitoring (ABPM)

ABPM is a non-invasive examination in which multiple, indirect, oscillometric, blood pressure measurements are obtained over a 24-hour period. The readings were performed every 20 minutes in the period from 7 am to 10p.m. and every 30 minutes in the period from 10p.m. to 7a.m.

Before starting the test, the mercury sphygmomanometer calibration was performed, and the cuff and recording device (TM-2430, A & D Company, Tokyo, Japan) were placed on the patient’s arm. The patient was instructed to keep a diary regarding his routine during the examination day, such as time of any physical activity, sleep and wake time, symptomatology, and medications in use. All the readings were performed by the same cardiologist, who was blinded regarding the post-treatment status of the patient.

The variables analysed were as follows: mean systolic (PS) and diastolic (PD) pressures during the referred sleep and wakefulness periods and during the 24 hours of monitoring and the decrease in systolic and diastolic pressures during sleep. A drop in the blood pressure during sleep, known as the nocturnal dip, of at least 10% was considered normal.

### Lateral pharyngoplasty

All patients underwent our lateral pharyngoplasty procedure, named version 4 (in the first 10 cases) and version 5 (in the following 7 cases). No other concomitant surgeries were performed. All patients underwent general anaesthesia and orotracheal intubation. The surgery (version 4) consists of tonsillectomy, resection of supratonsillar tissue for better exposure of the lateral pharyngeal wall, division of the palatopharyngeus muscle from the superior pharyngeal constrictor, caudal myotomy of the palatopharyngeus muscle with preservation of its mucosal lining, myotomy of the remaining superior constrictor muscle at the posterior wall, inside the tonsillar fossa, with identification and preservation of the stylopharyngeus muscle, suturing of tonsil pillars with tensioning, and displacement of the soft palate forward and placement of fibrin glue inside the dead space between the tonsil pillars. The following technical differences were included in version 5 of the surgery: the myotomy of the superior constrictor muscle was limited to 1cm in its cranial portion, the suturing of the tonsil pillars did not intend to displace the soft palate forward (tension free), and a vertical relaxation incision was added on each side, dividing each palatopharyngeus muscle from the posterior pharyngeal wall, which advances the soft palate. The palatopharyngeus muscle remained attached to the pharynx inferiorly, through its mucosal lining.

As a criterion for surgical success, we considered AHI reduction by more than 50% of baseline with a final AHI of less than 20/h.

### Statistical analysis

The Statistical Package for Social Sciences (SPSS Inc., Chicago, IL, USA), version 17 - 14.0 for Windows, was used for the elaboration of the database and descriptive analysis. The results are presented through tables and graphs. Categorical variables are expressed as frequencies and percentages. Continuous variables with normal distribution are expressed as the means and standard deviations; and those with non-normal distribution are expressed as medians and interquartile ranges. The normality of the numerical variables was verified through descriptive statistics, graphical analysis and the Shapiro-Wilk test.

For paired analysis of anthropometric, clinical, polysomnographic and blood pressure variables, the paired t-test was used for the parametric variables and the Wilcoxon test for the non-parametric variables. For all statistical analysis, a significance level of 5% (*p*<0.05) was considered.

The study would require a sample size of 21 patients to achieve a power of 80% and a level of significance of 5% (two sided), for detecting a mean difference of 0.10mm in CIMT, assuming the standard deviation of the differences to be 0.15mm.

## RESULTS

The number of patients in the study was 17, including 10 women and 7 men. The mean age was 45.9±8.8 years, ranging from 30 to 58 years. All patients were overweight (BMI>25kg/m^2^) before surgery, with a mean BMI of 30.0±2.2kg/m^2^, ranging from 25.2 to 36.2kg/m^2^. Eight patients (47.1%) had severe OSA, 3 patients (17.6%) had moderate OSA, and 6 patients (35.3%) had mild OSA, with a mean AHI of 22.6 (13.6-61.4). Regarding pharyngeal anatomy, 8 patients (47.1%) were classified as Friedman stage II and 9 patients (52.9%) as Friedman III. We identified 7 patients (41.2%) with one or more comorbidities that could increase CIMT. Hypertension was present in 4 cases, diabetes in 2 and dyslipidaemia in one; all of them were being treated with medication, which was maintained throughout the study. Four patients were smokers, maintaining this habit in the postoperative period.

After the surgeries, there were small but statistically significant reductions in weight and BMI (Table 1). The cervical and abdominal circumferences did not significantly change. There were significant decreases in the snoring intensity (*p*<0.001) and in the Epworth sleepiness scale (*p*=0.001) (Table 1).

**Table 1:** Pre- and postoperative anthropometric and clinical data.

Parameter	Preoperative	Postoperative	p-value
Weight, kg (mean±SD)	84.7 ± 13.9	82.8 ± 13.5	0.02*
BMI, kg/m^2^ (mean±SD)	30.0 ± 2.2	29.3 ± 2.3	0.03*
Cervical circumference, cm (mean±SD)	40.0 ± 3.4	39.6 ± 3.9	0.36
Abdominal circumference, cm (mean±SD)	103.2 ± 7.9	101.7 ± 6.6	0.10
Snoring intensity (median, IQR)	10 (9-10)	3 (1-4)	<0.001*
Epworth Sleepiness Scale (median, IQR)	15 (9-17.5)	5 (2-10)	0.001*

**Notes:** BMI = Body mass index; SD = Standard deviation; IQR = Interquartile range;

* *p*<0.05.

Postoperative polysomnography was performed an average of 7 months after the surgical procedure, ranging from 6 to 13 months. We found significant improvements in AHI, apnoea index, arousal index and minimum oxyhaemoglobin saturation after the surgeries. There was also a tendency for an increase in the mean nocturnal oxyhaemoglobin saturation (Table 2). A total of 76.4% of the patients were considered surgical successes.

**Table 2:** Pre- and postoperative polysomnographic data.

Parameter	Preoperative	Postoperative	p-value
AHI (median, IQR)	22.6 (13.6-61.4)	5.9 (1.8-24.4)	<0.001*
AI (median, IQR)	15.6 (4.6-47.2)	3.0 (0.3-7.2)	<0.001 *
Arousal index (median, IQR)	35.2 (22.2-55.6)	14.4 (7.5-27.3)	0.001*
Mean O_2_ (mean±SD)	91.5 ± 4.9	93.7 ± 2.5	0.08
Minimum O_2_ (mean±SD)	78.5 ± 9.5	83 ± 10.2	0.002*
T90% (median, IQR)	3.2 (0.8-17.7)	0.8 (0-5.3)	0.21

**Notes:** AHI = Apnoea-hypopnoea index; AI = Apnoea index; Mean O_2_ = Mean oxyhaemoglobin saturation; Minimum O_2_ = Minimum oxyhaemoglobin saturation; T90% = Percentage of total sleep time with oxyhaemoglobin saturation < 90%; IQR = Interquartile range; SD = Standard deviation;

* *p*<0.05.

Carotid ultrasonographies were performed an average of 7 months after the surgeries. Only three of our patients presented abnormal thickening of the carotid intima-media layer (CIMT>0.9mm) preoperatively. There was no significant difference in the CIMT after the surgeries (Table 3).

**Table 3:** Pre- and postoperative carotid ultrasonography.

Parameter	Preoperative	Postoperative	p-value
CIMT, right carotid, mm (mean±SD)	0.67 ± 0.14	0.72 ± 0.15	0.13
CIMT, left carotid, mm (mean±SD)	0.70 ± 0.14	0.70 ± 0.14	0.78

Notes: CIMT = Carotid intima-media thickness; SD = Standard deviation.

ABPM was performed an average of 7 months postoperatively. There was no significant difference in the blood pressure measurements after the surgeries (Table 4). Only one patient presented elevated blood pressure measurements preoperatively, despite the use of anti-hypertensive drugs.

**Table 4:** Pre- and postoperative ambulatory blood pressure monitoring data.

Blood pressure, mmHg	Preoperative	Postoperative	p-value
Systolic BP, 24 h (mean±SD)	122.7 ± 8.1	125.0 ± 10.7	0.19
Diastolic BP, 24 h (mean±SD)	76.5 ± 5.6	78.7 ± 6.6	0.15
Systolic BP, awake (mean±SD)	126.3 ± 8.5	128.1 ± 10.4	0.35
Diastolic BP, awake (mean±SD)	80.0 ± 6.4	81.0 ± 7.2	0.53
Systolic BP, sleep (mean±SD)	111.1 ± 12.7	110.4 ± 12.3	0.85
Diastolic BP, sleep (mean±SD)	67.0 ± 8.3	67.6 ± 8.5	0.83
Systolic BP, dip% (median, IQR)	12.5 (7.3-17.8)	14.0 (11.0-16.8)	0.70
Diastolic BP, dip% (median, IQR)	13.5 (9.3-18)	17.0 (15.0-20.5)	0.24

**Notes:** BP = Blood pressure; SD = Standard deviation; IQR = Interquartile range.

## DISCUSSION

In this small prospective study, despite a marked improvement in OSA, there was no significant variation in CIMT 6 months after the surgeries. Although this represents a negative outcome, we believe this study builds on previous knowledge and helps us define an optimal timing for CIMT evaluation after OSA treatment. It is not clear yet when CIMT may decrease after treatment of OSA and how CIMT may evolve if OSA is left untreated. Previous studies found no significant reduction in CIMT in OSA patients after 3 months of CPAP^15,22^. Four months of CPAP also did not seem to decrease CIMT in a large group of OSA patients^23^, but it decreased CIMT in a smaller group^14^. Another study showed that, after a 2-year follow-up, CIMT decreased in OSA patients treated with CPAP but did not evolve in OSA patients who were left untreated^17^. This last study included a group of patients with similar CIMT to our patients (mean, 0.68±0.12mm in the CPAP-treated group), emphasizing that, even in patients with CIMT within the normal range, OSA treatment may be expected to reduce CIMT in the long run.

A recent meta-analysis failed to demonstrate that CPAP would decrease CIMT among OSA patients, although subgroup analysis indicated a decrease in CIMT among patients with AHI≥50 and in those with CPAP use ≥6 months^16^. Severity of OSA, according to the AHI, may^12^ or not^17^ be associated with increased CIMT. Severity of minimum oxyhaemoglobin saturation seems to be a better predictor of increased CIMT^24,25^.

The early onset of atherosclerosis is usually evaluated by means of its subclinical signs, mainly by using carotid ultrasonography to evaluate CIMT because this is a widely accessible, non-invasive, low-cost technique^26^. Measurements of vascular wall thickness suffer significant variation according to the technique used and region examined^27^. As reflected in our study, the common carotid arteries represent the habitual site chosen for measuring CIMT^28^. One study found significant reductions in the intima-media thickness of the internal carotid (not common carotid) arteries after six months of concomitant uvulopalatopharyngoplasty and nasal surgeries^18^. That studied group had a mean AHI of 64.2, which is much higher than that in our group. Another study evaluating 53 patients who underwent uvulopalatopharyngoplasty found improvement in parameters of arterial stiffness but not in CIMT 6 months after surgery^18^.

Our patients achieved significant improvements in polysomnographic and clinical parameters, including a 10-point drop in the Epworth sleepiness scale and decreased snoring complaints. The surgical success rate in this study was 76.4%. However, there was no significant improvement in the 24-hour blood pressure measures at least 6 months after the procedures. In a previous study from our group, we reported that lateral pharyngoplasty significantly reduced blood pressure in OSA patients during sleep and during a 24-h period after a 6-month follow-up^29^. In this previous study, with a surgical success rate of 50%, patients underwent our lateral pharyngoplasty version 3 with significant improvements in AHI and in <90% oxyhaemoglobin sleep time. Regarding the differences in surgical technique, in lateral pharyngoplasty versions one^30^, two^31,32^, and three, there was no intent to advance the soft palate but only to enlarge the pharynx laterally. As of version 4 (the current study used versions 4 and 5), lateral pharyngoplasty additionally displaced the soft palate forward. In an attempt to explain the lack of response regarding the blood pressure in our current study - despite better polysomnographic outcomes - we reviewed our previous publication^29^, and the studied groups were comparable, except the baseline blood pressures were lower in our current study.

Obesity is considered to be the major risk factor for OSA and weight loss usually leads to an improvement in OSA. Some authors suggest that an at least 10% weight loss would have an impact on AHI^33^. In our study, despite the statistically significant variation in BMI, we observed that this reduction was very low (on average, 2kg in weight), and all patients remained overweight postoperatively. We can then assume that this small weight reduction should not have had a significant impact on AHI and that the pharyngeal reconstruction was responsible for the OSA improvement in our patients.

The findings of our study are limited due to our small sample size and the lack of a control group. One meta-analysis could not identify any influence of the sample size (<20 or ≥20) on the impact of CPAP on CIMT^16^. Regarding the imaging exams, the ultrasound analysis was done by only one physician in our study what could be considered a limitation. Another limitation of our study is that we did not have many participants with an AHI above 50, which is the threshold where patients got a decrease in CIMT after 6 months of CPAP, in a meta-analysis^16^. Other than OSA, 41.2% of our patients presented one or more risk factors for increased CIMT, such as smoking, hypertension, diabetes, and dyslipidaemia, which could have counterbalanced the potential beneficial impact of surgery over CIMT. In addition, a follow-up of 6 months may not be long enough to identify a decrease in CIMT following OSA treatment. Finally, 52.9% of our patients had mild or moderate OSA, which may have contributed to undermining the impact of OSA treatment on the early signs of atherosclerosis.

## CONCLUSION

Notwithstanding a significant improvement in OSA after lateral pharyngoplasty, there was no significant reduction in CIMT over a 6-month follow-up.

## References

[1] Leung RS, Bradley TD (2001). Sleep apnea and cardiovascular disease. Am J Respir Crit Care Med.

[2] Marin JM, Carrizo SJ, Vicente E, Agusti AGN (2005). Long-term cardiovascular outcomes in men with obstructive sleep apnoea-hypopnoea with or without treatment with continuous positive airway pressure: an observational study. Lancet.

[3] Valham F, Mooe T, Rabben T, Stenlund H, Wiklund U, Franklin KA (2008). Increased risk of stroke in patients with coronary artery disease and sleep apnea: a 10-year follow-up. Circulation.

[4] Marin JM, Artal J, Martin T, Carrizo SJ, Andres M, Martin-Burriel I (2014). Epigenetics modifications and subclinical atherosclerosis in obstructive sleep apnea: the EPIOSA study. BMC Pulm Med.

[5] Yokoe T, Minoguchi K, Matsuo H, Oda Net, Minogushi H, Yoshino G (2003). Elevated levels of C-reactive protein and interleukin-6 in patients with obstructive sleep apnea syndrome are decreased by nasal continuous positive airway pressure. Circulation.

[6] Somers VK, Dyken ME, Clary MP, Abboud FM (1995). Sympathetic neural mechanisms in obstructive sleep apnea. J Clin Invest.

[7] Savransky V, Nanayakkara A, Li J, Bevans S, Smith PL, Rodriguez A (2007). Chronic intermittent hypoxia induces atherosclerosis. Am J Respir Crit Care Med.

[8] Chambless LE, Heiss G, Folsom AR, Rosamond W, Szklo M, Sharrett AR (1997). Association of coronary heart disease incidence with carotid arterial wall thickness and major risk factors: the Atherosclerosis Risk in Communities (ARIC) Study, 1987-1993. Am J Epidemiol.

[9] Yeboah J, Redline S, Johnson C, Tracy R, Ouyang P, Blumenthal RS (2011). Association between sleep apnea, snoring, incident cardiovascular events and all-cause mortality in an adult population: MESA. Atherosclerosis.

[10] Lurie A. (2011). Obstructive sleep apnea in adults. Adv Cardiol.

[11] Farooqui FA, Sharma SK, Kumar A, Soneja M, Mani K, Radhakrishnan R (2017). Endothelial function and carotid intima media thickness in obstructive sleep apnea without comorbidity. Sleep Breath.

[12] Drager LF, Bortolotto LA, Lorenzi MC, Figueiredo AC, Krieger EM, Lorenzi-Filho G (2005). Early signs of atherosclerosis in obstructive sleep apnea. Am J Respir Crit Care Med.

[13] Gunnarsson SI, Peppard PE, Korcarz CE, Barnet JH, Aeschlimann SE, Hagen EW (2014). Obstructive sleep apnea is associated with future subclinical carotid artery disease: thirteen-year follow-up from the Wisconsin sleep cohort. Arterioscler Thromb Vasc Biol.

[14] Drager LF, Bortolotto LA, Figueiredo AC, Krieger EM, Lorenzi GF (2007). Effects of continuous positive airway pressure on early signs of atherosclerosis in obstructive sleep apnea. Am J Respir Crit Care Med.

[15] Ng SS, Liu EK, Ma RC, Chan TO, To KW, Chan KK (2017). Effects of CPAP therapy on visceral fat thickness, carotid intima-media thickness and adipokines in patients with obstructive sleep apnoea. Respirology.

[16] Chen LD, Lin L, Lin XJ, Ou YW, Wu Z, Ye YM (2017). Effect of continuous positive airway pressure on carotid intima-media thickness in patients with obstructive sleep apnea: a meta-analysis. PLoS One.

[17] Català R, Ferré R, Cabré A, Girona J, Porto M, Texidó A (2016). Efecto a largo plazo del tratamiento con presión positiva continua de la vía aérea sobre la arteriosclerosis subclínica en el síndrome de apnea-hipopnea durante el sueño. Med Clin (Barc).

[18] Peng Y, Zhang L, Hu D, Dai Y, Wang S, Liao H (2016). Reduction of internal carotid artery intima-media thickness in patients with moderate-to-severe obstructive sleep apnea syndrome after nasal surgery and uvulopalatopharyngoplasty. Acta Otolaryngol.

[19] Zhan X, Li L, Wu C, Chitguppi C, Huntley C, Fang F (2019). Effect of uvulopalatopharyngoplasty (UPPP) on atherosclerosis and cardiac functioning in obstructive sleep apnea patients. Acta Otolaryngol.

[20] Friedman M, Vidyasagar R, Bliznikas D, Joseph N (2005). Does severity of obstructive sleep apnea/hypopnea syndrome predict uvulopalatopharyngoplasty outcome?. Laryngoscope.

[21] Johns MW (1991). A new method for measuring daytime sleepiness: the Epworth sleepiness scale. Sleep.

[22] Kostopoulos K, Alhanatis E, Pampoukas K, Georgiopoulos G, Zourla A, Panoutsopoulos A (2016). CPAP therapy induces favorable short-term changes in epicardial fat thickness and vascular and metabolic markers in apparently healthy subjects with obstructive sleep apnea-hypopnea syndrome (OSAHS). Sleep Breath.

[23] Kim J, Mohler ER, Keenan BT, Maislin D, Arnardottir ES, Gislason T (2017). Carotid artery wall thickness in obese and nonobese adults with obstructive sleep apnea before and following positive airway pressure treatment. Sleep.

[24] Gunnarsson SI, Peppard PE, Korcarz CE, Barnet JH, Hagen EW, Hla KM (2015). Minimal nocturnal oxygen saturation predicts future subclinical carotid atherosclerosis: the Wisconsin sleep cohort. J Sleep Res.

[25] Baguet JP, Hammer L, Lévy P, Pierre H, Launois S, Mallion JM (2005). The severity of oxygen desaturation is predictive of carotid wall thickening and plaque occurrence. Chest.

[26] Toth PP (2008). Subclinical atherosclerosis: what it is, what it means and what we can do about it. Int J Clin Pract.

[27] Ho SSY (2016). Current status of carotid ultrasound in atherosclerosis. Quant Imaging Med Surg.

[28] Touboul PJ, Hennerici MG, Meairs S, Adams H, Amarenco P, Bornstein N (2007). Mannheim carotid intima-media thickness consensus (2004-2006). An update on behalf of the Advisory Board of the 3rd and 4th Watching the Risk Symposium, 13th and 15th European Stroke Conferences, Mannheim, Germany, 2004, and Brussels, Belgium, 2006. Cerebrovasc Dis.

[29] Soares CFP, Cavichio L, Cahali MB (2014). Lateral pharyngoplasty reduces nocturnal blood pressure in patients with obstructive sleep apnea. Laryngoscope.

[30] Cahali MB (2003). Lateral pharyngoplasty: a new treatment for obstructive sleep apnea hypopnea syndrome. Laryngoscope.

[31] Cahali MB, Friedman M (2009). Sleep apnea and snoring: surgical and non-surgical therapy.

[32] Mesti Junior J, Cahali MB (2012). Evolution of swallowing in lateral pharyngoplasty with stylopharyngeal muscle preservation. Braz J Otorhinolaryngol.

[33] Peppard PE, Young T, Palta M, Dempsey J, Skatrud J (2000). Longitudinal study of moderate weight change and sleep-disordered breathing. JAMA.

